# Acknowledgment to Reviewers of *Audiology Research* in 2021

**DOI:** 10.3390/audiolres12010009

**Published:** 2022-01-27

**Authors:** Audiology Research Editorial Office

**Affiliations:** MDPI AG, St. Alban-Anlage 66, 4052 Basel, Switzerland

Rigorous peer-reviews are the basis of high-quality academic publishing. Thanks to the great efforts of our reviewers, *Audiology Research* was able to maintain its standards for the high quality of its published papers. Thanks to the contribution of our reviewers, in 2021, the median time to first decision was 21 days and the median time to publication was 57.5 days. The editors would like to extend their gratitude and recognition to the following reviewers for their precious time and dedication, regardless of whether the papers they reviewed were finally published:
Adam SheppardJosée LagacéAgnieszka J. SzczepekKatarina Trebusak PodkrajsekAgnieszka Janik McErleanKiran K. SriperumbudurAlessandra FiorettiKoji NishimuraAlexander J. BilligLuca BruschiniAli A. DaneshLuca VerrecchiaAllegra CattaniMarcello CherchiAnke TropitzschMarwan El MobadderAugusto CasaniMatias MorinBenjamin BoeckingMelissa TesherChiara VisentinMike MaslinChip HahnMin Young LeeChristian ByaertMing ZhangChristian GiguèreMónica ChamorroChristopher PastrasNicolas Perez-FernandezCristiano BalzanelliPasqua Irena SciancaleporeDaniel B. PolleyPasqualina Maria PicciottiDelia HorhatPeter V. PaulDominika OziębłoRichard C. DowellEkaterina GarbarukRobert J. MorellEleonora M.C. TreccaRoberto TeggiEliane SchochatRuggero LapennaFabrice GiraudetSaba BattelinoFaris F. BrkicSalvatore MartellucciFei ZhaoSamue AtchersonFrancesca AtturoSergio Cuevas-CovarrubiasFrancesco GaziaSeyed Alireza RohaniGeorge TavartkiladzeShunsuke KidaniGerrit PaascheSimona SacchiniGiampiero NeriSung Kwang HongGiuseppe AttanasioTang-Chuan WangHenning SchepkerToshihisa MurofushiHero P. WitVijaya Kumar NarneHiroshi Inui InuiVincenzo De CeglieIan CurthoysVirginia CorazziInna BelyantsevaWieslaw KonopkaJan de LaatWiktor JedrzejczakJerzy TomikXiying GuanJohannes FellingerYael ZaltzJose Antonio Lopez-Escamez



